# Physiological Conditions Leading to Maternal Subclinical Ketosis in Holstein Dairy Cows Can Impair the Offspring’s Postnatal Growth and Gut Microbiome Development

**DOI:** 10.3390/microorganisms11071839

**Published:** 2023-07-19

**Authors:** Jessica Halfen, Nathaly Ana Carpinelli, Sergio Lasso-Ramirez, Tainara Cristina Michelotti, Emily C. Fowler, Benoit St-Pierre, Erminio Trevisi, Johan S. Osorio

**Affiliations:** 1 School of Animal Science, Virginia Tech, Blacksburg, VA 24061, USA; halfenj@vt.edu; 2 Department of Dairy and Food Science, South Dakota State University, Brookings, SD 57007, USA; nathalyanacarpinelli@gmail.com (N.A.C.); lassoram@ualberta.ca (S.L.-R.); tainara.michelotti@inrae.fr (T.C.M.); 3 Deparment of Agricultural, Food and Nutritional Science, University of Alberta, Edmonton, AB T6G 2R3, Canada; 4 Unité Mixte de Recherche sur les Herbivores, INRAE, F-63122 Saint-Genès-Champanelle, France; 5 Department of Animal Science, South Dakota State University, Brookings, SD 57007, USA; emily.fowler@sdstate.edu (E.C.F.); benoit.st-pierre@sdstate.edu (B.S.-P.); 6 Department of Animal Sciences, Food and Nutrition (DIANA), Faculty of Agriculture, Food and Environmental Science, Università Cattolica del Sacro Cuore, 29122 Piacenza, Italy; erminio.trevisi@unicatt.it

**Keywords:** calves, ketosis, gut microbiome, 16S rRNA, bacterial composition

## Abstract

Maternal metabolic disruptions, such as ketosis, can have adverse effects on fetal development and influence postnatal factors. Twelve Holstein calves were randomly enrolled in this study at birth and monitored until 8 weeks of age. The study was conducted from fall 2018 until spring 2019. After completing the data collection period, calves were classified according to their respective dams ketotic condition after parturition. This classification was based on dam blood β-hydroxybutyrate < 1.4 mmol/L nonketotic (NONKET; n = 6 calves) or ≥1.4 mmol/L subclinical-ketotic (SK; n = 6 calves). SK calves had greater birth body weight (*p* = 0.05) but exhibited a slower growth rate compared to NONKET calves from 1 to 8 weeks (*p* = 0.02). At birth, SK calves had lower (*p* < 0.01) levels of non-esterified fatty acids and bilirubin compared to NONKET calves. Analysis of feces alpha diversity indicates that by 3 weeks, NONKET calves had greater diversity, richness, and evenness. *Butyricicoccus pullicaecorum* and *Gallibacterium anatis* were more abundant in SK calves (*p* < 0.05) at 3 weeks. In contrast, NONKET calves had a greater (*p* < 0.05) abundance of *Sharpae azabuensis* at 3 weeks. These findings suggest that subclinical ketosis in cows can impact the in-utero development, postnatal growth, and maturing gut microbiome of their offspring.

## 1. Introduction

Ketosis is a metabolic disorder commonly observed in dairy cows during the peripartal period, characterized by high concentrations of ketone bodies in the blood. The incidence of ketosis in dairy cows is estimated to be at least 40%, depending on various factors such as environment, parity, and nutrition, among others [1]. This condition is associated with reduced feed intake, decreased milk production, and increased risk of other health disorders [2], as well as immunometabolic changes prior to calving [3].

Maternal stress prior to calving can have significant implications for the offspring’s growth and development. The fetus relies entirely on the dam for nutrients during gestation, and any metabolic disturbances in the dam can affect fetal development and impact postnatal growth, feed efficiency, and gut health [4]. In late gestation, maternal stressors can alter the supply of nutrients to the fetus through the umbilical cord [5]. Maternal stressors, including energy intake and over-conditioning, have been correlated with changes in birth BW [6,7], which can have substantial implications for postnatal growth [7,8]. Additionally, birth-related complications or stressors can shift the normal concentration of potent metabolic and physiological regulators such as glucocorticoids [9]. It has been observed that dystocia at calving can decrease the normal secretion of fetal glucocorticoids and diminish the natural maturation of enterocytes soon after calving [9], consequently impairing the vascularization of enterocytes and subsequently the absorption of immunoglobulins and bioactive compounds in colostrum [10].

In ruminants, it has been demonstrated that maternal nutrition significantly impacts the offspring’s gut development [11]. Similar to humans, bacteria have been detected in newborn calf meconium [12] and bovine uterus [13], suggesting that the maternal microbiome can have an effect on fetal gut colonization [14]. Furthermore, maternal stressors such as anxiety and depression can affect the gut microbiome in infants leading to lower microbial taxa involved in maintaining proper brain and immune function [15]. However, in dairy cows, little is known about the impact of maternal stressors during late gestation on postnatal colonization and development of the gut microbiota.

Previous data on the gut microbiome in the pre-weaned calf indicate that bacterial communities are simple and less diverse at birth. These communities then increase in complexity and diversity as the calf grows due to age and dietary changes [16]. Additionally, microbial taxa detected in stool samples taken from pre-weaned calves have shown pattern changes (i.e., increasing or decreasing) in their relative abundance as the animal developed. In particular, certain bacterial genera, including *Bifidobacterium*, *Lactobacillus*, *Fecalibacterium*, and *Enterococcus,* have been shown to decrease in abundance as the calf grows [17,18,19].

Although the effects of ketosis on the dam have been extensively characterized, less is known about the impact on the offspring. We hypothesized that physiological alterations in late gestation leading to maternal ketosis postpartum would affect calf growth, performance, and gut microbiome maturation during postnatal life. Therefore, the objective of this study was to evaluate the effects of physiological conditions leading to maternal subclinical ketosis in dairy cows on offspring growth and development, metabolism, immune response, and gut microbiome.

## 2. Materials and Methods

The Institutional Animal Care and Use Committee (IACUC) of South Dakota State University approved all the procedures for this study (protocol no. 18-028A).

### 2.1. Maternal Management

Details for the original experimental design to test the effects of yeast culture fermentation products fed to transition dairy cows have been published previously [20]. Briefly, 40 multiparous Holstein cows were enrolled in a randomized complete block design from −30 to 50 days in milk (DIM) and blocked according to expected calving day, parity, previous milk yield, and genetic merit. At −30 DIM, cows were assigned to a common closed-up diet [1.39 Mcal/kg of dry matter (DM) and 12.3% crude protein (CP)] plus 114 g/d of ground corn (control; n = 20) or plus 100 g/d of ground corn and 14 g/d of YC (n = 20), fed as a top-dress. Cows received the same lactation diet from calving to 50 DIM (1.60 Mcal/kg of DM and 15.6% CP). Cows were enrolled in the experiment from mid-September 2018 to early April 2019. Cows were fed using an individual gate system (American Calan, Northwood, NH, USA), and feed intakes were recorded daily. Diets were formulated using the Cornell Net Carbohydrate and Protein System model, contained in the NDS Professional ration formulation software (Nutritional Dynamic System; v. 6.55; RUM&N) to meet 100% of National Research Council (NRC) nutrient requirements [21]. During the dry period, cows were housed in a bedded pack pen, and 3 d before the expected calving date, cows were reallocated in individual pens bedded with straw until parturition. After parturition, cows were moved within 2 h to an individual chute and milked with a porta-milker vacuum pump (Nasco, Fort Atkinson, WI, USA; Cat. no. Z15664N). On d 3 postpartum, cows were moved to a free-stall barn. A retrospective analysis was performed in this subset of cows, using the postpartum β-hydroxybutyrate (BHB) data measured on whole blood collected before morning feeding at 1, 3, 5, 7, and 9 DIM with the precision Xtra (Abbott Diabetes Care Inc., Alameda, CA, USA). Cows with an average BHB < 1.4 mmol/L were classified as nonketotic (NONKET; n = 6), and cows with ≥1.4 mmol/L were classified as subclinical-ketotic (SK; n = 6). The mean BHB concentration for NONKET and SK was 0.59 ± 0.2 and 1.82 ± 0.3 mmol/L, respectively. On average, NONKET cows had 4.2 ± 0.4 time points below the subclinical ketosis threshold (1.4 mmol/L), and SK cows had 2.1 ± 1.5 time points above the threshold. The threshold used in this study was based on findings by Oetzel et al. [22]. The average prepartum BW and BCS for NONKET and SK cows are presented in Table S1.

### 2.2. Calf Enrollment Criteria

As the cows were giving birth, a subset of calves from each group (YC, n = 8; Control, n = 7) was randomly selected to be followed until weaning. After completing the data collection period (from 0 to 56 days of age), each calf was classified based on the respective dam’s ketotic condition. This classification resulted in 6 Holstein dairy calves classified as SK, and in order to match the same number of animals in the NONKET group, only 6 calves out of 9 NONKET calves were randomly selected. Calves enrolled in this study were born from October 2018 to April 2019. Variability of calf pairs was minimized by applying the following inclusion criteria: (1) calving difficulty score < 3, where 3 represents minimum assistance, but no calving difficulty, (2) calf birth weight ≥ 32 kg, (3) single calf, (4) colostrum quality ≥ 21% brix refractometer, and (5) 3.8 L of colostrum intake from the same dam. This criteria protocol is based on Johnson et al. [23] with modifications by Osorio et al. [24]. After birth, calves were weighed and measured, had the navel disinfected with a 7% tincture of iodine solution (First Priority Inc., Elgin, IL, USA), and vaccinated with TSV II (Pfizer Inc., New York, NY, USA) via nostril application, and received 3.8 L of first milking colostrum from the respective dam within 2 h after birth. Calves were offered first milking colostrum again on the second feeding at 4 h after birth if colostrum intake had not reached the 3.8 L required. The calves exhibited a natural sucking reflex, eliminating the need for tubing. Calves were housed in individual hutches bedded with straw and monitored from birth to 8 weeks of age. None of the animals enrolled in the study showed clinical signs of disease.

Calves were fed with 2.8 L of milk replacer (20% fat and 20% protein) twice a day from week 1 to week 6 and then switched to once a day until the weaning at week 8. The milk replacer was reconstituted at 12% solids of a commercial antibiotic-free milk replacer (Herd Maker^®^PB; Land O’Lakes Animal Milk products, Arden Hills, MN, USA). Calves had access to a starter grain mix (19.9% CP, 13.5% NDF) offered ad libitum from week 1 until the end of the study, and the intake was recorded daily. Health checks (fecal and respiratory scores) were recorded daily until weaning. For fecal scores, a classification scale of 1–4 was used (1: firm, well-formed (not hard); 2: soft, pudding-like; 3: runny, pancake batter; 4: liquid, splatters), and for respiratory scores, a scale of 1–5 was used (1 = normal to 5 = dry cough) [25]. Rectal temperature was recorded daily until 21 d of age. Growth performance, including body weight (BW) and withers height (WH), was recorded weekly. Average daily gain (ADG) was calculated by dividing the weekly weight gain by 7 (days).

### 2.3. Sample Collection

Blood samples were collected from the jugular vein using 20-gauge BD vacutainer needles before colostrum (baseline), 48 h after colostrum, and then at 2, 3, and 6 weeks of age. Samples were collected into evacuated serum tubes (BD Vacutainer, Becton Dickinson, Franklin Lakes, NJ, USA) containing either clot activator or lithium heparin for serum or plasma, respectively. After blood collection, tubes with sodium heparin were placed on ice, and tubes with clot activator were kept at room temperature until centrifugation (~30 min) at 1300× *g* for 15 min at 4 °C and 21 °C, respectively. Aliquots of serum and plasma were frozen (−80 °C) until further analysis for determination of blood metabolites, oxidative stress biomarkers, and acute-phase proteins (APP). Fecal samples were collected by finger stimulation with a sterile glove within 48 h post-colostrum feeding and then at 1 and 3 weeks of age. Fecal debris was removed from the perianal region with a paper towel before fecal sampling, and the first portion of the sample was discarded to avoid cross-contamination with the environment. The samples were placed in a cryovial and immediately frozen in liquid nitrogen at −80 °C until further microbiota analysis.

### 2.4. Blood Metabolites, APP, and Oxidative Stress Biomarkers

Blood samples were analyzed for glucose, cholesterol, urea, ceruloplasmin, albumin, creatinine, glutamic-oxaloacetic transaminase (GOT), γ-glutamyltransferase (GGT), bilirubin, haptoglobin (HP), ferric reducing antioxidant power (FRAP), paraoxonase (PON), myeloperoxidase (MPO), and total reactive oxygen metabolites (ROM) using kits purchased from Instrumentation Laboratory (IL Test). Non-esterified fatty acids (NEFA) and BHB were measured using kits from Wako Chemicals (Mountain View, CA, USA) and Randox Laboratories Ltd. (Crumlin, Northern Ireland, UK), respectively, following the procedures described previously [26,27] using a clinical auto-analyzer (ILAB 650, Instrumentation Laboratory, Lexington, MA, USA). Bovine interleukin-6 (IL-6) plasma concentration was determined by a colorimetric sandwich ELISA using a Bovine IL-6 Screening Set (#ESS0029 Endogen, Pierce, Rockford, IL, USA).

### 2.5. Fecal DNA Extraction, 16S rRNA Gene Amplification, and Sequencing

Total DNA was isolated from fecal samples using the QIAamp DNA mini kit (Qiagen, Hilden, Germany) according to methods described by Rosa et al. [28]. Briefly, 0.2 g of feces were mixed with 1 mL of buffer InhibitEX in a 2-mL RNasefree O-ring tube containing one stainless steel bead, 5 mm (Qiagen, Hilden, Germany), and then homogenized in a Beadbeater (BioSpec Products, Bartlesville, OK, USA) for 30 s. Following homogenization, samples were kept on ice for 1 min, then centrifuged at 20,000× *g* for 1 min at 20 °C. After centrifugation, 600 µL of supernatant was transferred to a new microcentrifuge tube containing 25 µL of Qiagen Proteinase K, followed by the addition of 600 µL of Buffer AL. The mixture was vortexed for 15 s, then incubated at 70 °C for 10 min in a heat block. After incubation, 600 µL of 96% molecular ethanol was added and vortexed. The mixture was transferred into a QIAamp mini spin column, and the subsequent steps were performed according to the manufacturer’s procedures (Qiagen, Hilden, Germany). DNA quantity and purity were measured using a Nanodrop spectrophotometer (ND 1000, Nanodrop Technologies, Inc., Wilmington, DE, USA).

MiSeq 2 × 300 paired-end reads from amplicons targeting the V1–V3 region of the bacterial 16S rRNA gene were generated by the University of Minnesota Genomics Center. Overlapping raw forward and reverse reads from the same flow cell clusters were assembled into contigs using the ‘make.contigs’ command from MOTHUR (v 1.44) [29]. Unless specified otherwise, all other sequence data analysis steps were performed using custom-written Perl scripts. V1–V3 contigs were then selected to meet the following criteria: the presence of both intact 27F (forward) and 519R (reverse) primer nucleotide sequences, a length between 400 and 580 nt, and an average Phred quality score of Q33 or greater.

Following quality screens, sequence reads were aligned, then clustered into Operational Taxonomic Units (OTUs) at a genetic distance cutoff of 4% sequence dissimilarity [30,31]. While 3% is the most commonly used clustering cutoff for 16S rRNA, it was originally recommended for full-length sequences and may not be suitable for the analysis of specific sub-regions since nucleotide sequence variability is not constant across the entire length of the 16S rRNA gene. In this context, if 3% is a commonly accepted clustering cutoff for V4 or V4–V5 regions, which are the least variable of the hypervariable regions, then a higher cutoff should be used for the V1–V3 region since V1 is the most variable region of the 16S rRNA gene [32,33].

OTUs were screened for DNA sequence artifacts using the following methods. Chimeric sequences were first identified with the ‘chimera.uchime’ [34] and ‘chimera.slayer’ [35] commands from the MOTHUR (v 1.44.1) open-source software package. Secondly, the integrity of the 5′ and 3′ ends of OTUs was evaluated using a database alignment search-based approach; when compared to their closest match of equal or longer sequence length from the NCBI (National Center for Biotechnology Information) ‘nt’ database, as determined by BLAST [36], OTUs with more than five nucleotides missing from the 5′ or 3′ end of their respective alignments were discarded as artifacts. Single-read OTUs were subjected to an additional screen, where only sequences that had a perfect or near-perfect match to a sequence in the NCBI ‘nt’ database were kept for analysis, i.e., the alignment had to span the entire sequence of the OTU, and a maximum of 1% of dissimilar nucleotides was tolerated.

After the removal of sequence chimeras and artifacts, taxonomic assignment of valid OTUs was performed. All OTUs were classified at the levels of Phylum and Family using RDP Classifier [37]. For select OTUs, BLAST queries were also used to identify their respective closest valid relatives [36]. Alpha diversity indices (Observed OTUs, Chao, Ace, Shannon, and Simpson) were determined using the ‘summary.single’ command from MOTHUR (version 1.44.1) [29] on a dataset rarified to 5000 reads for each sample. Principle Coordinate Analysis (PCoA) for beta diversity was performed using the same rarefied dataset by first determining Bray Curtis distances with the ‘summary.shared’ command, followed by the ‘pcoa’ command in MOTHUR (version 1.44.1) [29].

### 2.6. Statistical Analysis

Data were analyzed with the Proc MIXED procedure of SAS 9.4 (SAS Institute Inc., Cary, NC, USA). Fixed effects in the model were group (G), Time (T, day or week), and their interaction (G × T). The random effect was a calf within a group. The authors acknowledge that the low sample size (n = 6/group) of this study could have impaired the statistical power. However, the stringent criteria applied to enrolled calves in this study were necessary to reduce additional noise in our data. Additionally, this type of maternal-neonatal experiment is difficult to perform; therefore, a similar low sample size has been used in other studies [24,38]. Repeated measured data for BW, WH, ADG, starter intake, fecal score, respiratory score, and rectal temperature were modeled, selecting the variance-covariance structures with the least Bayesian information criterion value among compound symmetry, autoregressive, or heterogeneous autoregressive. The exponential correlation covariance structure SP for repeated measures was used to analyze blood metabolites. Comparisons of abundance for bacterial taxonomic groups and OTUs amongst G × T were performed in R (Version R-3.6.2), first using the nonparametric test Kruskal–Wallis, followed by pairwise comparisons with the Wilcoxon test, which included the Benjamini–Hochberg correction for multiple comparisons. Blood metabolites and fecal microbial abundance results were log_2_-scale transformed if needed to comply with the normal distribution of residuals and subsequently back-transformed. The least squares mean separation between time points was performed using the PDIFF statement. Statistical significance was declared at *p* ≤ 0.05 and tendencies at *p* ≤ 0.10.

## 3. Results

### 3.1. Growth Performance and Health

**Figure 1:**
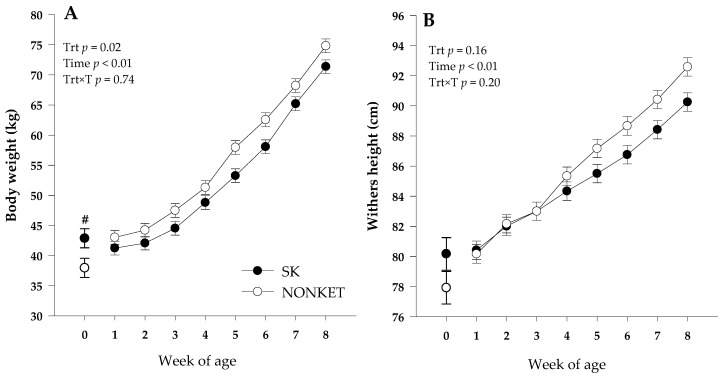
Body weight (Panel (**A**)) and withers height (Panel (**B**)) from 1 to 8 weeks of Holstein dairy calves born to dams classified as subclinical ketotic (SK; ≥1.4 mmol/L) or nonketotic (NONKET; <1.4 mmol/L) during the first 10 days postpartum. Body weight and withers height at birth was included at week 0, and only birth body weight was different between groups (#; *p* = 0.05). The *p*-values for the main effect of group (G), time (T), and their interaction (G × T) are shown. Values are means, and SE are represented by vertical bars.

Although a greater birth BW (*p* = 0.05) was observed for SK calves compared with NONKET, there was a trend (*p* = 0.06) for greater final BW in the NONKET group compared with SK. The latter effect could be attributed to the overall greater (*p* = 0.02) BW in NONKET calves from birth to 8 weeks of age. This effect can be associated with a slower growth rate in SK calves than NONKET from 1 to 8 weeks (Figure 1A). Even though there was no effect (*p* = 0.17) on birth WH, a trend (*p* = 0.06) for higher final WH in NONKET calves compared with SK was observed, with no overall WH effect (*p* = 0.16; Figure 1B). On average, NONKET calves had a lower (*p* = 0.05) number of days on scours (7 vs. 2.8 days) compared to SK calves. Maternal predisposition to postpartal subclinical ketosis did not affect offspring ADG, starter intake, fecal score, respiratory score, and rectal temperature (*p* ≥ 0.15; Table 1).

### 3.2. Blood Biomarkers

**Figure 2:**
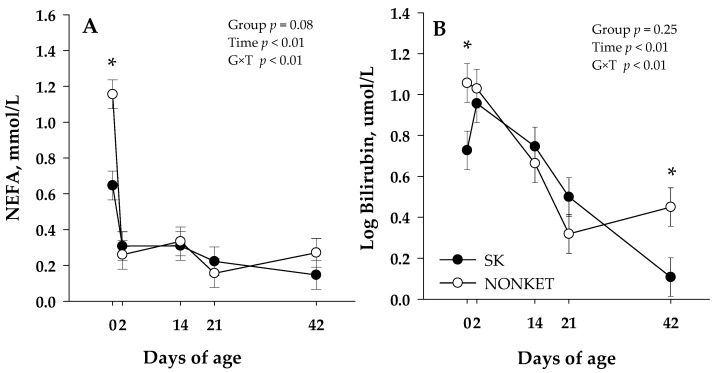
Blood non-esterified fatty acid (NEFA; Panel (**A**)) and bilirubin (Panel (**B**)) from calves born to dams classified as subclinical ketotic (SK; ≥1.4 mmol/L) or nonketotic (NONKET; <1.4 mmol/L) during the first 10 days postpartum. Data are from samples taken at birth (precolostrum; 0 d), 2 d (postcolostrum), 14, 21, and 42 d of age. The *p*-values for the main effect of group (G), time (T), and their interaction (G × T) are shown. Main separation between time points was evaluated when a G × T interaction (*p* ≤ 0.10) was observed, and differences (*) were declared at *p* ≤ 0.05. Values are means, and SE are represented by vertical bars.

Blood biomarkers were not affected by G × T interaction except for NEFA (*p* < 0.01) and bilirubin (*p* < 0.01) (Table 2). Additionally, a trend on G × T was observed for urea (*p* = 0.10), albumin (*p* = 0.09), and ceruloplasmin (*p* = 0.06). The G × T in NEFA was related to a lower (*p* < 0.01) NEFA in SK calves than NONKET at 0 d (Figure 2A); in turn, this effect was further associated with a trend (*p* = 0.08) for an overall lower NEFA in SK calves than NONKET. Similar to NEFA, the G × T effect in bilirubin was associated with greater (*p* = 0.02) bilirubin in NONKET calves than SK at 0 d; however, at 42 d, NONKET calves have lower (*p* = 0.01) bilirubin than SK (Figure 2B). The trend for G × T in albumin was attributed to a lower (*p* = 0.02) albumin concentration in NONKET calves than SK at 14 d (Figure S1A). The trend for G × T in urea was related to a lower (*p* = 0.02) urea concentration in NONKET calves than SK at 2 d (Figure S1B). The trend for G × T in ceruloplasmin was associated with lower (*p* = 0.05) ceruloplasmin in NONKET calves than SK at 14 d, followed by greater (*p* = 0.01) ceruloplasmin in NONKET calves than SK at 42 d (Figure S1C). Glucose, BHB, creatinine, cholesterol, GOT, GGT, haptoglobin, IL-6, paraoxonase, ROM, FRAP, and MPO were not affected by group effects.

### 3.3. Fecal Bacterial Community Composition

**Figure 3:**
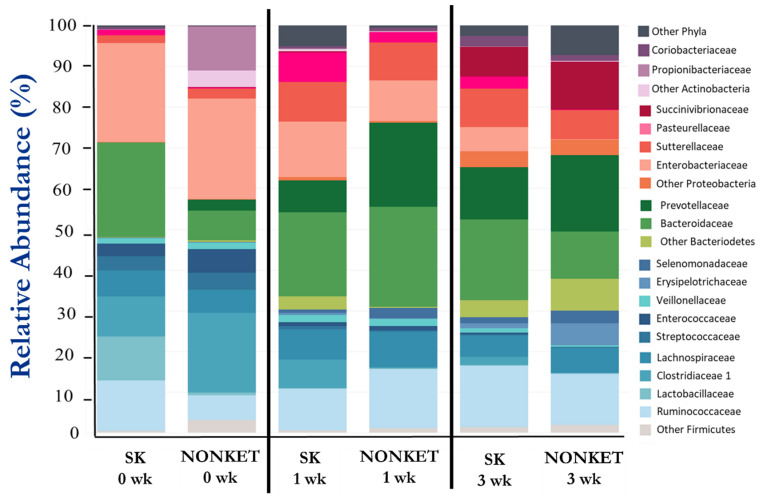
Fecal bacterial composition from calves born to dams classified as subclinical ketotic (SK; ≥1.4 mmol/L) or nonketotic (NONKET; <1.4 mmol/L) during the first 10 days postpartum. Data are from fecal samples taken within 48 h postcolostrum feeding (0 weeks), then at 1 and 3 weeks of age. Abundance is presented as a percentage (%) of the total number of analyzed reads per sample.

A total of 292,156 high-quality-filtered sequence reads from V1-V3 amplicons of the 16S rRNA were used for bacterial composition analysis. The mean relative abundance (%) of the main bacterial taxonomic groups is presented in Table 3 and Figure 3, with Firmicutes, Bacteroidetes, and Proteobacteria representing the most prominent phyla in this study. While fluctuations in the abundance of certain families were observed amongst groups and time points, statistical support for differences in taxonomic composition was found for only Eryspelotrichaceae, with lower abundance in the SK group compared to NONKET at 3 weeks (*p* < 0.05).

**Figure 4:**
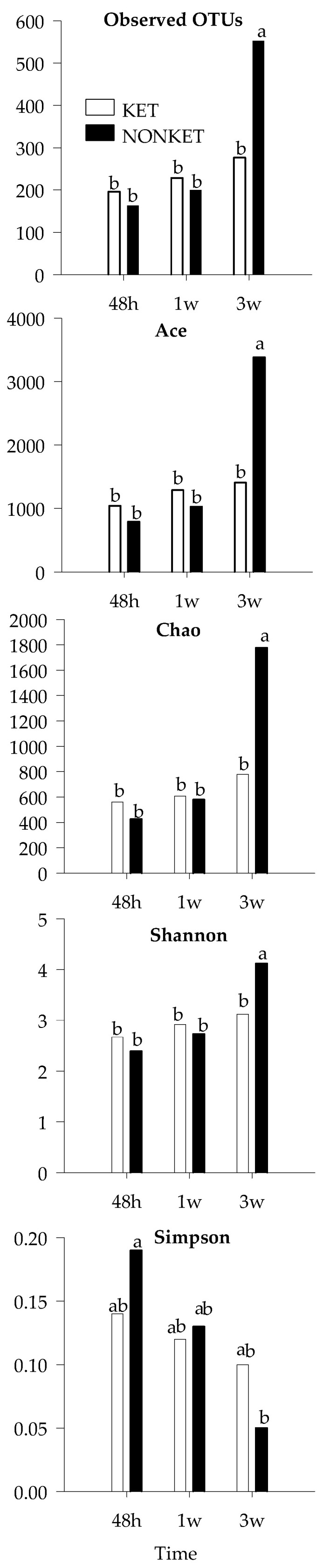
Mean values for α-diversity indices in fecal samples derivate from dairy calves born to dams classified as subclinical ketotic (SK; ≥1.4 mmol/L) or nonketotic (NONKET; <1.4 mmol/L) during the first 10 days postpartum. Statistical differences by ANOVA (*p* < 0.05). ^ab^ Different superscripts indicate that groups are significantly different by Tukey’s range test for multiple pairwise comparisons.

To gain further insight, an analysis based on OTU composition was performed. While beta diversity analysis showed no discernable clustering according to group or timepoint (Figure S4), the alpha diversity indices ‘observed OTUs’, ‘Ace’, and ‘Chao’ indicated that the number of bacterial species was at least 2× higher in NONKET at 3 weeks compared to any other group (*p* < 0.05; Figure 4). While showing a more modest difference, the Shannon index was also found to be higher in NONKET at 3 weeks (*p* < 0.05). In light of these observed variations in composition, the most abundant OTUs were further analyzed with the goal of identifying potential bacterial species that were differentially represented between the two groups of calves (Table 4). Notably, three OTUs were found to differ in abundance between SK and NONKET at 3 weeks. The abundance of Bt-1197 (*Butyricicoccus pullicaecorum*) and of Bt-1238 (*Gallibacterium anatis*) were greater in SK calves at 3 weeks (*p* < 0.05), with levels that were 12.8× and 44.0× higher, respectively, than in the NONKET samples from the same time point. Bt-1391 (*Sharpae azabuensis*) showed an opposite pattern, with levels that were 3.0× higher in NONKET samples compared to SK at 3 weeks.

## 4. Discussion

### 4.1. Growth Performance and Health

Evidence that maternal diet, health status, and environment during late gestation in dairy cows can affect the development and performance of the offspring has been previously shown [6,38,39]. However, the effects of prepartal metabolic alterations in dairy cows predisposed to postpartal subclinical ketosis on the offspring’s growth and performance have not been investigated.

Prior data have associated prenatal exposure to metabolic stress [38], heat stress [40,41], and high maternal body condition score [7] with lower birth BW. Interestingly, in the current study, we observed a greater birth BW in calves born to dams programmed to develop subclinical ketosis postpartum. It is plausible that while SK cows had a lower negative energy balance (NEB) before calving due to lower dry matter intake (DMI), the flux of nutrients to the fetus was maintained. This allowed the fetus to develop normally at the expense of a worsened maternal immunometabolic status during the days leading to calving. Although SK calves had greater birth BW, they grew at a lower rate, reflected in lower final BW (69.4 vs. 74.1) and shortest final WH (90.4 vs. 92.4) in SK calves than NONKET. The slower growth rate in SK calves could be partially attributed to the greater number of days on scours (fecal score = 4; 7 vs. 2.8) during the entire study. The latter is in agreement with prior literature on other mammalian models. For instance, Sussman et al. [42] used a mouse model to evaluate the effects of a gestational ketogenic diet on offspring development, and they observed retarded physiological growth as well as alterations to neonatal brain structure.

Ling et al. [38] evaluated the carryover effects of maternal metabolic stress caused by high prepartal lipid mobilization (>0.3 mmol/L NEFA) on the offspring’s growth, metabolism, and immune response. In contrast to our results, Ling et al. [38] observed that calves born to dams under high prepartal lipid mobilization had a lower birth BW than control calves. Similarly to the current study, they also reported a slower growth rate from birth to 4 weeks of age in calves born to cows under high prepartal lipid mobilization. This effect was associated with a compromised immune response due to similarities with calves born to dams exposed to heat stress during late gestation, which included low BW at birth and weaning coupled with immune suppression [40]. The correlation between NEFA and BHB blood concentration in dairy cows during the transition period is well established [43]. During this period, cows enter a NEB condition due to the increased energy requirements and lower DMI. This leads to a large mobilization of fatty acids stored in adipose tissue, thus, causing marked elevations in blood concentrations of NEFA and, consequently, BHB, thereby inducing ketosis [44]. In the current study, SK cows had a contrasting greater NEFA (*p* = 0.08; Figure S2) and lower DMI (*p* = 0.08; Figure S3) compared to NONKET cows, which describes a greater lipolysis in SK cows. However, NEFA effects were mostly related to postpartal lipolysis rather than prepartum. Results from Alharthi et al. [7] partially agree with our findings, where lower postnatal growth was observed in calves born to dams with a high BCS in late gestation (BCS ≥ 3.75) and low DMI during the last 4 weeks of pregnancy. Therefore, the low postnatal growth observed in SK calves could be associated with low maternal DMI prepartum because no effects (*p* = 0.41; Table S1) were observed in prepartal BCS between SK and NONKET cows. Additionally, Alharthi et al. [7] observed lower birth BW in calves born to dams with high prepartal BCS, contrasting with the greater birth BW observed in SK calves compared to NONKET.

The effects on BW and WH between SK and NONKET calves were seen in the present study and in previous research on prenatal exposure to metabolic stress by Alharthi et al. [7]. Heat stress [40] and maternal BCS [38] reinforce the hypothesis that maternal stress during late pregnancy can imprint permanent development conditions in the offspring. Likewise, previous data in ruminants and non-ruminant species have shown that maternal stress during gestation influences fetal development and exerts carryover effects on the offspring due to a suboptimal intrauterine condition [45,46]. Therefore, the reduction in postnatal BW observed in SK calves indicates that maternal metabolic programming leading to postpartal ketosis potentially altered in-utero development and postnatal growth parameters in the offspring; however, more research should focus on the specific mechanism for these effects.

### 4.2. Blood Biomarkers

The newborn has to withstand a brief period of starvation soon after birth before receiving a new source of nutrients from the colostrum. During this period, the calf depends entirely on mobilizing glycogen and fat stores for survival [47]. Therefore, metabolic processes such as lipolysis, glycogenolysis, and gluconeogenesis must be quickly activated after birth to maintain normal blood glucose concentration [48]. Alharthi et al. [7] observed lower NEFA at birth for calves born to dams with a high prepartal BCS (BCS ≥ 3.75) in comparison to low BCS cows (≤3.25); however, no overall effect was observed in NEFA throughout the rest of the trial. These data agree with the current study, where low NEFA was observed at birth in SK calves compared to NONKET. Ling et al. [38] found no effect on NEFA concentration in calves born to dams with high NEFA during late gestation. The greater NEFA concentration observed in NONKET calves indicates a more developed metabolism ready to use fat as an energy source. Additionally, high NEFA at birth before receiving colostrum may indicate a better extra-uterine adaptation in NONKET calves since brown adipose tissue in newborns utilizes NEFA as fuel for thermoregulation [49]. This suggests that the prepartal physiological conditions leading to ketosis in SK cows may impair offspring’s adaptation to an extra-uterine environment.

Although there was a greater bilirubin concentration in NONKET when compared with SK calves at birth (11.38 vs. 5.33 µmol/L) and at 42 d (2.81 vs. 1.28 µmol/L), the levels at both time points were within the range observed by other authors in Holstein dairy calves from 1 d to 12 days [50] and from 1 d to 84 d [51]. The higher bilirubin level around 24 and 48 h of life has been related to fetal hemoglobin destruction and lower excretion of bilirubin [50]. Similarly, Alharthi et al. [7], evaluating the effect of maternal BCS, reported a trend for greater plasma bilirubin at birth on calves born to dams with low BCS. This effect was translated into overall greater bilirubin for the same group of calves, which the authors associated with a more developed metabolism. Macrophages are the primary cells responsible for the uptake and disposal of heme, which is an important function in preventing cellular toxicity from heme and iron-mediator, recycling iron to sustain erythropoiesis, and preventing pathogen’s access to iron during infection [52]. Heme is a component of hemoglobin that is phagocytosed by macrophages and monocytes when it reaches the end of its lifespan. Heme is then released during this process and converted to biliverdin, rapidly converted to bilirubin, and then transported to the liver [52]. Therefore, it is conceivable that the calves born to NONKET dams in the present study were born with a more developed macrophage system capable of performing tissue-specific homeostatic functions.

Acute-phase proteins (APP) are produced during an acute-phase response, mostly associated with an inflammation or infection process [53,54]. The APPs can be classified as positive (increase) or negative (decrease) depending on their change in blood concentration during an inflammatory condition [54]. For example, albumin is a negative APP, while ceruloplasmin is a positive APP [54]. Although there was a trend for lower albumin concentration at 14 d in NONKET calves compared to SK (23.2 vs. 28.5 g/L), the levels in both groups were within similar ranges for a 14-d old calf [51]. Rosa et al. [55] observed a maximal ceruloplasmin concentration in Jersey calves undergoing mild diarrhea during 2 to 3 weeks of age. In agreement with Rosa et al. [55], Hajimohammadi et al. [53] also observed greater ceruloplasmin levels in Holstein dairy calves undergoing diarrhea within 1 day to 4 months old. Together, these data suggest that differences between SK and NONKET calves in ceruloplasmin at 14 and 42 d of age might be due to an inflammatory condition. While SK had the greatest ceruloplasmin at 14 d (2.37 vs. 1.95 µmol/L), NONKET calves had greater ceruloplasmin at 42 d (1.97 vs. 1.42 µmol/L), indicating, perhaps, transient bouts of inflammation in both groups. However, it is important to emphasize that these levels are lower than those observed by Rosa et al. [55] when calves suffered from mild diarrhea, indicating that calves in the current study were under lesser physiological stress.

### 4.3. Fecal Bacterial Community Composition

There is a large body of evidence on how maternal stress influences fetal development and its postnatal consequences. For instance, fetal intestinal development and neonatal gut microbial colonization can be affected by many prenatal factors, such as maternal stress conditions (e.g., heat stress and malnutrition), maternal microbiota, and colostrum quality [16]. Osorio [56] recently reviewed the alterations in intestinal mass and length, villus and crypt density, and their negative effects on passive immune transfer in different neonatal species due to different maternal stressor conditions. However, the effects of a metabolic disorder such as ketosis during the late-gestation period on the gut microbial environment of a newborn calf remains unclear.

Erysipelotrichaceae is a family comprising of anaerobic, facultatively anaerobic, or aerobic bacteria of the order Erysipelotrichales and the Firmicutes phylum that can utilize a variety of substrates for their metabolism, including carbohydrates, lipids, and amino acids [57]. Members of the Erysipelotrichaceae have been associated with metabolic disorders and inflammatory diseases of the animal host [57,58]. Interestingly, Huang [59] compared gut microbiomes between healthy and ketotic cows and observed a lower abundance of Eryspelotrichaceae in ketotic cows. The latter aligns with the lower abundance of Eryspelotrichaceae in SK calves compared to NONKET at 3 weeks of age. This suggests that maternal programming from cows predisposed to develop ketosis can be passed onto their offspring’s developing gut microbiome, and such effects seem to be amplified over time; however, the actual mechanism for this effect remains to be elucidated.

In a recent study, fecal microbial changes were evaluated across age and diarrhea effects in dairy calves, where a decrease in Enterobacteriaceae abundance was observed over time from birth to 5 weeks [60]. This agrees with the decrease in Enterobacteriaceae abundance observed in NONKET calves at 3 weeks compared to 48 h. This effect was also confirmed by a lower number of scour days in NONKET calves compared to SK. According to Kim et al. [60], the high abundance during the pre-weaning period might have been introduced directly to newborn calves through the feces of the dam. Following this argument, Huang [59] observed that fecal samples from ketotic cows showed a higher Enterobacteriaceae abundance than control cows. Therefore, although there is no evidence in the present study that NONKET cows experienced a lower fecal abundance of this taxa, it is plausible that there might be an underlying factor leading to a quicker decrease in Enterobacteriaceae abundance over time (0 vs. 3 weeks) in NONKET calves. In addition, Gomez et al. [61] reported that dairy calves experiencing diarrhea have a characteristic shift in gut bacteria from obligate anaerobes to facultative anaerobes and an increased abundance of Enterobacteriaceae along with Enterococcaceae. The decrease of both taxa in NONKET calves from 48 h compared with 3 weeks could indicate a healthier intestinal microbiota in NONKET calves compared with SK.

Moreover, the presence of taxa associated with volatile fatty acid metabolism, such as Selenomonadaceae and Succinivibrionaceae, could also promote a better energy source for the host. Ruminal Selenomonadaceae and Succinivibrionaceae taxa have been shown to actively metabolize succinate into propionate [62,63]. Additionally, Succinivibrionaceae have also been suggested to use H_2_ as a substrate for the production of succinate or propionate, acting as an alternative sink for H_2_ instead of methane synthesis by ruminal archaea [64]. Therefore, it is possible that the increase in Selenomonadaceae and Succinivibrionaceae abundance from 48 h to 3 weeks in NONKET calves but not in SK may indicate better fermentation and nutrient utilization in calves born to nonketotic dams. However, the potential metabolic and physiological roles of these taxa in the gut, which is suggested by their presence in fecal microbial communities, remain to be further studied in dairy calves as well as in ruminants in general.

The abundance of OTU Bt-1238, predicted to be a strain of *Gallibacterium anatis*, a bacterial species that has been associated with diarrhea in calves [61], was lower in NONKET calves at 3 weeks compared to 48 h. *G. anatis* has been associated with increased mortality in poultry and conditions such as respiratory problems, liver necrosis, and hemorrhagic peritonitis, among others [65], as well as bronchopneumonia in calves [66]. While there were no statistically supported differences between the two groups of calves, OTUs Bt-1063 and Bt-1343 were also affiliated with bacterial species known to include pathogenic strains. For instance, *Escherichia coli* (Bt-1063) has been associated with diarrhea in calves [61]. While prior literature on the pathogenicity of *Enterococcus cecorum* (Bt-1343) in dairy calves or ruminants is scarce, Dolka et al. [67] reported that this species was the main culprit for enterococcal spondylitis in chickens. A disease previously known as enterococcal vertebral osteoarthritis. *E. cecorum* has also been found to be an opportunistic pathogen in humans, as it plays a role in the etiology of nosocomial infections [67,68].

Other OTUs of interest were predicted to be strains of beneficial bacterial species. Indeed, Bt-1197 was affiliated with *Butyricicoccus pullicaecorum*, a butyrate producer with probiotic properties [69], while Bt-1391 was predicted to be a strain of *Sharpae azabuensis*, which may be involved in ruminal fatty acid metabolism, such as linoleic and linolenic acid [70]. While the numerical increase in abundance for OTUs Bt-1112 and Bt-1452 could not be resolved statistically in this study, their affiliation to *Succinivibrio dextrinosolvens* was of great interest. Indeed, members of the *Succinivibrio* genus are known to ferment glucose into end products such as acetic and succinic acids. Notably, a few strains of *S. dextrinosolvens* have been shown to possess the necessary enzymes to degrade and assimilate nitrogen-containing compounds [71].

During the preruminant phase, the small and large intestines serve as the main sites for digestion and absorption of nutrients. Therefore, beneficial gut microbial colonization is necessary to maintain an adequate host’s nutrient supply, mucosal immune system, and overall health [72,73]. In this context, several studies have found a positive correlation between gut microbial diversity and the host’s health status and development. For instance, decreased gut microbial diversity has been reported in diarrheic Holstein dairy calves, suggesting a possible link between gut microbiota and host health [61,74]. Additionally, Oikonomou et al. [17] observed an increased microbial diversity coupled with increased BW gain in healthy dairy calves, which aligns with the present study results. Therefore, the increased alpha diversity indexes, including Ace, Chao, and Shannon in NONKET calves compared to SK at 3 weeks, indicates that NONKET calves had a greater microbial diversity and evenness than SK. Since Ace and Chao’s indexes estimate the number of undetected species in a sample, while Shannon takes into account both species richness and evenness, this suggests that by 3 weeks of age, NONKET calves had more species present in their gut microbiome and the relative abundance of these species was more even than in SK calves. Additionally, the lower Simpson index in NONKET calves indicates that their gut microbiome community was less dominated by a few species and that the abundance of different species was more evenly distributed. Taken together, these results support the idea that late-gestation maternal ketogenic condition affects the offspring’s microbiome gut.

## 5. Conclusions

The results from this study highlight the impact of offspring in-utero exposure to maternal conditions leading to ketosis postpartum. Maternal stress during late pregnancy, including lower DMI, caused greater birth BW but lower postnatal growth in SK calves coupled with greater scour days. This reinforces the hypothesis that maternal metabolic programming leading to postpartal ketosis potentially altered in-utero development and postnatal growth parameters in the offspring. At birth, the lower NEFA and bilirubin in SK calves indicated a less developed metabolism and preparedness for an extra-uterine environment when thermoregulation is key before receiving colostrum. This scenario may have also affected SK calves’ ability to absorb vital nutrients and immunoglobulins in colostrum. The fecal microbiome in SK calves revealed that these calves had a greater abundance of pathogenic taxa and bacteria coupled with lower taxa and bacteria correlated with better nutrient utilization and metabolism. Analysis of alpha diversity indexes suggests that by 3 weeks of age, NONKET calves had a greater diversity, richness, and evenness. However, the actual mechanism for these effects remains to be elucidated. Therefore, further research is needed to investigate the impact of maternal programming and ketosis on dairy calves’ metabolism, immunity, and gut microbiome.

## Figures and Tables

**Table 1:** Growth performance and health parameters of calves born to dams classified as subclinical ketotic (SK; ≥1.4 mmol/L) or nonketotic (NONKET; <1.4 mmol/L) during the first 10 days postpartum.

Item	Group ^1^		SEM ^2^	*p*-Value		
	SK	NONKET		Group (G)	Time	G × T ^3^
BW, kg	53.16	56.19	0.76	0.02	<0.01	0.74
Birth BW, kg	42.86	37.95	1.59	0.05	-	-
Final BW, kg	69.43	74.11	1.47	0.06	-	-
WH, cm	85.08	86.19	0.49	0.16	<0.01	0.20
Birth WH, cm	80.17	77.92	1.08	0.17	-	-
Final WH, cm	90.36	92.36	0.66	0.06	-	-
ADG, kg/d	0.55	0.62	0.04	0.18	<0.01	0.39
Starter intake, kg/d	0.55	0.64	0.05	0.21	<0.01	0.40
Fecal scores	2.06	1.81	0.12	0.15	<0.01	0.98
Fecal score > 3 (d)	7.0	2.8	1.3	0.05	-	-
Respiratory score	1.13	1.19	0.05	0.44	0.53	0.76
Rectal temperature, °C	38.46	38.44	0.10	0.88	0.11	0.58

^1^ Calves borne from cows classified as subclinical-ketotic (n = 6, SK; >1.4 mmol/L) or non-ketotic (n = 6, NONKET; <1.4 mmol/L). Cow classification was based on whole blood BHB concentration at 1, 3, 5, 7, 9, and 11 days postpartum. ^2^ Largest SEM. ^3^ G × T = interaction of group × time (week).

**Table 2:** Blood biomarkers of metabolism, liver function, inflammation, and oxidative stress parameters of calves born to dams classified as subclinical ketotic (SK; ≥1.4 mmol/L) or nonketotic (NONKET; <1.4 mmol/L) during the first 10 days postpartum.

Parameter	Group ^1^		SEM ^2^	*p*-Value		
	SK	NONKET		Group (G)	Time	G × T ^3^
Metabolites and liver function						
Glucose, mmol/L	5.41	5.46	0.26	0.88	<0.01	0.53
NEFA, mmol/L	0.33	0.44	0.04	0.08	<0.01	<0.01
BHB, mmol/L	0.08	0.08	0.00	0.95	<0.01	0.33
Creatinine, μmol/L	98.12	97.05	4.74	0.88	<0.01	0.42
Cholesterol, mmol/L	1.59	1.62	0.17	0.90	<0.01	0.18
Urea, mmol/L	4.03	3.81	0.17	0.37	0.02	0.10
Albumin, g/L	27.17	25.83	0.97	0.20	0.01	0.09
GOT, U/L	51.62	50.73	3.08	0.84	<0.01	0.21
Log GGT, U/L	2.04	1.97	0.09	0.57	<0.01	0.59
Bilirubin, μmol/L	1.52	1.63	0.06	0.25	<0.01	<0.01
Inflammation						
Ceruloplasmin, μmol/L	1.35	1.41	0.08	0.58	<0.01	0.06
Haptoglobin, g/L	0.30	0.28	0.01	0.42	0.05	0.27
IL-6, pg/mL	192.2	220.25	30.31	0.53	0.14	0.83
Paraoxonase, U/mL	33.80	32.08	3.01	0.69	<0.01	0.15
Oxidative stress						
ROM, mg H_2_O_2_/100 mL	13.08	14.74	0.80	0.17	<0.01	0.15
FRAP, μmol/L	144.92	136.92	6.19	0.38	<0.01	0.58
Myeloperoxidase, U/L	285.8	283.2	18.35	0.92	<0.01	0.96

^1^ Calves borne from cows classified as subclinical-ketotic (n = 6, SK; >1.4 mmol/L) or non-ketotic (n = 6, NONKET; <1.4 mmol/L). Cow classification was based on whole blood BHB concentration at 1, 3, 5, 7, 9, and 11 days postpartum. ^2^ Largest SEM. ^3^ G × T = interaction of group × time (week).

**Table 3:** Mean relative abundance (%) of main bacterial taxonomic groups in fecal samples of calves born to dams classified as subclinical ketotic (SK; ≥1.4 mmol/L) or nonketotic (NONKET; <1.4 mmol/L) during the first 10 days postpartum.

Taxonomic Affiliation	0 Weeks		1 Week		3 Weeks		*p*-Value
	SK	NONKET	SK	NONKET	SK	NONKET	
** Firmicutes **	47.97	46.89	30.19	26.31	28.37	30.01	0.21
Ruminococcaceae	12.38	6.08	10.27	12.53	15.18	12.64	0.22
Lachnospiraceae	6.36	5.64	7.44	7.47	5.15	6.19	0.76
Clostridiaceae ^1,#^	9.92 ^c^	19.59 ^abc^	6.94 ^bc^	0.23 ^ab^	2.03 ^abc^	0.14 ^a^	0.04
Enterococcaceae ^#^	3.14 ^a^	5.73 ^a^	0.92 ^a^	1.00 ^a^	0.41 ^ab^	0.00 ^b^	0.01
Selenomonadaceae ^#^	0.00 ^b^	0.01 ^b^	0.74 ^ab^	2.26 ^ab^	1.57 ^ab^	3.16 ^a^	0.04
Erysipelotrichaceae ^#^	0.03 ^a^	0.06 ^a^	0.51 ^a^	0.07 ^a^	1.22 ^a^	5.41 ^b^	0.01
Veillonellaceae	1.49	1.81	1.83	1.50	0.95	0.43	0.62
Streptococcaceae	3.44	4.21	0.86	0.21	0.39	0.06	0.05
Lactobacillaceae	10.81	0.63	0.14	0.08	0.10	0.03	0.51
Other Firmicutes ^&^	0.41	3.13	0.54	0.97	1.36	1.94	-----
** Proteobacteria **	27.66	27.73	31.66	19.23	29.59	22.90	0.66
Enterobacteriaceae ^#^	24.28 ^c^	24.63 ^ac^	13.65 ^ac^	8.55 ^a^	5.83 ^abc^	0.08 ^b^	<0.01
Sutterellaceae	1.86	2.42	9.60	7.98	9.48	7.21	0.12
Succinivibrionaceae ^#^	0.00 ^a^	0.04 ^a^	0.21 ^a^	0.09 ^a^	7.22 ^ab^	11.70 ^b^	<0.01
Pasteurellaceae	1.48	0.46	7.39	2.23	3.03	0.11	0.06
Other Proteobacteria ^&^	0.03	0.19	0.81	0.37	4.04	3.80	-----
**Bacteriodetes** ^#^	23.41 ^b^	10.30 ^b^	31.80 ^ab^	38.92 ^a^	36.76 ^ab^	38.12 ^ab^	0.04
Bacteroidaceae	23.34	7.35	20.75	21.02	19.77	11.58	0.33
Prevotellaceae	0.04	2.66	7.81	17.72	12.80	18.70	0.07
Other Bacteroidetes ^&^	0.04	0.30	3.24	0.15	4.20	7.84	-----
** Actinobacteria **	0.58	4.89	1.23	0.81	2.69	1.73	0.41
Propionibacteriaceae	0.02	10.77	0.06	0.02	0.01	0.00	0.13
Coriobacteriaceae	0.54	0.09	0.52	0.76	2.53	1.39	0.12
Other Actinobacteria ^&^	0.02	4.03	0.65	0.04	0.16	0.33	-----
Other Bacteria ^&$^	0.05	0.02	0.64	0.06	0.32	0.89	-----

Note: Mean relative abundance of taxonomic groups is presented as a percentage (%) of the total number of analyzed reads per sample. ^1^ Calves borne from cows classified as subclinical-ketotic (n = 6, SK; >1.4 mmol/L) or non-ketotic (n = 6, NONKET; <1.4 mmol/L). Cow classification was based on whole blood BHB concentration at 1, 3, 5, 7, 9, and 11 days postpartum. ^#^ Taxa showing a statistically significant difference by the Kruskal–Wallis sum rank test (*p* < 0.05). Different superscript letters in the same row indicate that groups are significantly different by the Wilcoxon test for multiple pairwise comparisons (*p* < 0.05). ^&^ Statistical test not performed because of group heterogeneity. ^$^ Other bacteria include Fusobacteria, Campilobacterota, Verrucomicrobia, Elusimicrobia, Lentisphaerae, Spirochaetes, and Synergistetes, as well as unclassified bacteria.

**Table 4:** Mean relative abundance of the main bacterial OTUs identified from calves born to dams classified as subclinical ketotic (SK; ≥1.4 mmol/L) or nonketotic (NONKET; <1.4 mmol/L) during the first 10 days postpartum. Abundance is presented as a percentage (%) of the total number of analyzed reads per sample.

OTUs	0 Weeks		1 Week		3 Weeks		*p*-Value	Closest Valid Taxon (ID%)
	SK	NONKET	SK	NONKET	SK	NONKET		
** Proteobacteria **								
SD_Bt-1063 ^#^	25.30 ^a^	21.78 ^a^	19.98 ^a^	13.16 ^a^	9.52 ^ab^	0.09 ^b^	0.01	* Escherichia coli * (100%)
SD_Bt-1238 ^#^	1.34 ^ab^	<0.01 ^a^	7.91 ^b^	2.58 ^ab^	3.96 ^b^	0.09 ^a^	0.02	* Gallibacterium anatis * (96.39%)
SD_Bt-1112	0.00 *	0.01	0.17	0.09	4.54	8.50	0.06	* Su. dextrinosolvens * (96.88%)
SD_Bt-1452 ^#^	0.00 *^,a^	0.03 ^a^	0.04 ^a^	0.02 ^a^	4.49 ^ab^	5.63 ^b^	<0.01	* Su. dextrinosolvens * (96.89%)
SD_Bt-1319	0.02	0.69	2.21	0.58	0.67	0.57	0.89	* Pa. excrementihominis * (99.02%)
SD_Bt-19374	0.01	0.00 *	0.02	<0.0	4.32	0.10	0.48	* Rhodospirillum rubrum * (88.41%)
SD_Bt-1419	0.00 *	0.00 *	2.44	0.19	0.35	0.04	0.09	* Ca. hyointestinalis * (98.12%)
SD_Bt-1150	0.09	2.87	0.0	0.04	0.07	<0.01	0.05	* Klebsiella pneumoniae * (99.62%)
** Firmicutes **								
SD_Bt-1197 ^#^	10.51 ^a^	6.35 ^ab^	4.34 ^a^	11.19 ^a^	7.32 ^a^	0.57 ^b^	0.03	* Bu. pullicaecorum * (96.54%)
SD_Bt-1192	5.94	10.32	4.05	0.08	1.59	0.0	0.12	* Cl. perfringens * (99.80%)
SD_Bt-1206	1.81	1.70	2.94	2.01	1.11	0.61	0.54	* Ruminococcus gnavus * (99.2%)
SD_Bt-1708	<0.01	<0.01	0.42	3.14	1.37	3.57	0.15	* Megamonas rupellensis * (98.36%)
SD_Bt-1427	0.28	2.68	3.10	1.48	0.83	0.11	0.34	* Cl. bolteae * (97.16%)
SD_Bt-1343 ^#^	1.25 ^a^	2.02 ^a^	0.79 ^a^	1.53 ^a^	0.64 ^ab^	0.01 ^b^	0.03	* Enterococcus cecorum * (99.81%)
SD_Bt-1517 ^#^	1.16 ^ab^	0.02 ^b^	0.72 ^ab^	3.57 ^a^	0.28 ^b^	<0.01 ^b^	0.04	* Cl. bolteae * (99.05%)
SD_Bt-1021	3.00	2.25	0.81	0.05	0.40	0.06	0.59	* Streptococcus equinus * (100%)
SD_Bt-1709 ^#^	2.02 ^a^	3.45 ^a^	0.27 ^ab^	0.22 ^a^	0.00 *^,b^	0.00 *^,b^	0.01	* Enterococcus faecium * (100%)
SD_Bt-1075	1.04	4.07	0.05	0.02	0.17	<0.01	0.10	* Cl. paraputrificum * (99.80%)
SD_Bt-1391 ^#^	0.00 *^,a^	0.02 ^a^	0.12 ^a^	0.04 ^a^	0.83 ^a^	2.46 ^b^	<0.01	* Sharpea azabuensis * (100%)
** Bacteriodetes **								
SD_Bt-1064	19.88	0.64	7.19	17.08	1.49	2.41	0.10	* Bacteroides fragilis * (99.81%)
SD_Bt-1246	0.01	0.02	4.37	7.16	2.72	4.97	0.18	* Prevotella stercorea * (96.18%)
SD_Bt-1205	0.01	<0.01	1.54	1.46	5.19	3.16	0.22	* Pr. timonensis * (89.64%)
SD_Bt-1208	0.01	0.02	2.03	0.01	3.13	5.31	0.12	* Prevotella stercorea * (99.05%)
SD_Bt-1070	0.01	0.03	0.84	6.67	1.46	1.37	0.27	* Prevotella copri * (97.92%)
SD_Bt-0966	<0.01	3.90	0.01	5.12	1.56	0.37	0.22	* Prevotella veroralis * (90.77%)
SD_Bt-1072	0.04	0.39	2.12	0.49	0.32	2.26	0.07	* Bacteroides uniformis * (100%)
SD_Bt-1085 ^#^	0.00 *^,a^	0.00 *^,a^	0.96 ^ab^	0.00 ^a^	1.88 ^ab^	2.17 ^b^	<0.01	* Duncaniella freteri * (82.8%)
SD_Bt-1695	0.01	0.92	0.18	0.87	1.90	0.59	0.04	* Bacteroides vulgatus * (95.63%)
** Actinobacteria **								
SD_Bt-1548	0.02	10.62	0.06	0.02	0.01	<0.01	0.24	* Cutibacterium acnes * (99.80%)
SD_Bt-1201	0.58	0.11	0.86	1.35	4.36	1.62	0.11	* Collinsella aerofaciens * (98.38%)
** Fusobacteria **								
SD_Bt-1456	0.00 *	0.20	0.96	0.20	1.65	2.31	0.34	* Fusobacterium varium * (99.8%)

* no reads were detected in any of the samples for this group. ^#^ OTUs showing a statistically significant difference by the Kruskal–Wallis sum rank test (*p* < 0.05). Different superscript letters in the same row indicate that groups are significantly different by the Wilcoxon test for multiple pairwise comparisons (*p* < 0.05). Abbreviations: *Bu* = *Butyricicoccus*; *Ca.* = *Campylobacter; Cl* = *Clostridium; Pa* = *Parasutterella; Pr* = *Prevotellamassilia; Su.* = *Succinivibrio*.

## Data Availability

Raw sequence data are available in NCBI Sequence Read Archive under Bioproject PRJNA994536.

## Supplementary Materials

The following supporting information can be downloaded at: https://www.mdpi.com/article/10.3390/microorganisms11071839/s1, Table S1: Body weight and BCS for NONKET and SK cows from −3 to −1 weeks relative to parturition; Figure S1: Blood albumin, urea, and ceruloplasmin from calves born to dams classified as subclinical ketotic (SK; ≥1.4 mmol/L) or nonketotic (NONKET; <1.4 mmol/L) during the first 10 days postpartum; Figure S2: Blood non-esterified fatty acids from cows classified as subclinical ketotic (SK; ≥1.4 mmol/L) or nonketotic (NONKET; <1.4 mmol/L) during the first 10 days postpartum; Figure S3: Prepartal dry matter intake from cows classified as subclinical ketotic (SK; ≥1.4 mmol/L) or nonketotic (NONKET; <1.4 mmol/L) during the first 10 days postpartum; Figure S4: Comparison of fecal bacterial communities in calves born to subclinical ketotic (SK) or non-ketotic (NONKET) cows at 0, 1, and 3 wk of age. PCoA was performed using a Bray-Curtis distance matrix. The x and y axes correspond to Principal Components 1 (PCo1) and 2 (PCo2).
