# Supplementary material for: Physiological Conditions Leading to Maternal Subclinical Ketosis in Holstein Dairy Cows Can Impair the Offspring’s Postnatal Growth and Gut Microbiome Development
Source: Microorganisms. 2023 Jul 19;11(7):1839. doi: 10.3390/microorganisms11071839 (PMC10383123; doi:10.3390/microorganisms11071839)
Supplement: Supplementary file 1 [file microorganisms-11-01839-s001.zip › microorganisms-2459138-supplementary.pdf]

SUPPLEMENTAL MATERIALS

Table S1. Body weight and BCS for NONKET and SK cows from -3 until 0 weeks relative to parturition.

Parameter	Group		SEM[1]	P-Value			
	NONKET	SK		Group	Week	G×W	Lactation
BW, kg	818.7	868.3	31.9	0.29	0.30	0.68	> 0.05
BCS[2]	3.1	3.2	0.13	0.59	0.32	0.28	> 0.05

[1] Largest SEM.

[2] Body condition score (scale 1 = thin to 5 = obese)

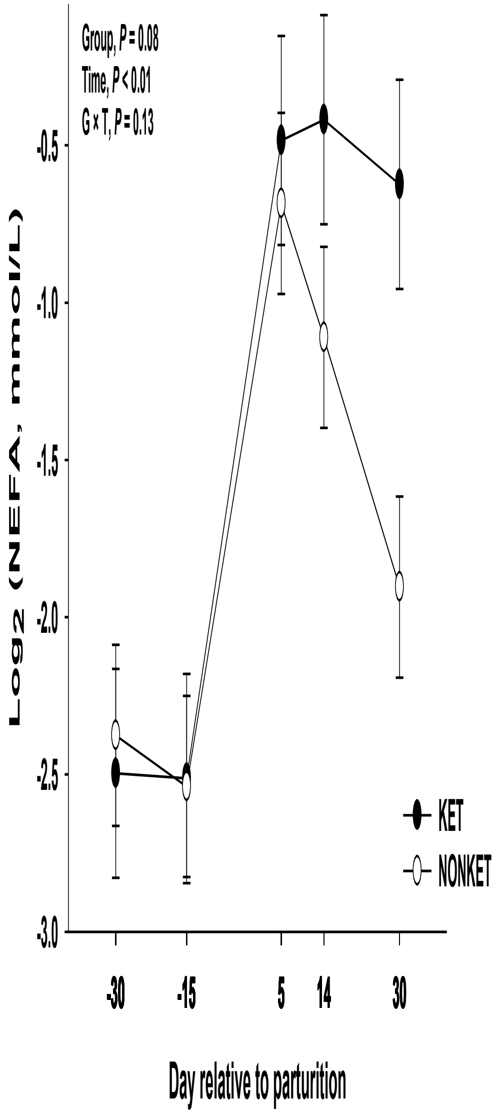

Figure S1. Blood albumin (Panel A), urea (Panel B), and ceruloplasmin (Panel C) from calves born to dams classified as subclinical ketotic (SK; ≥ 1.4 mmol/L) or nonketotic (NONKET; < 1.4 mmol/L) during the first 10 days postpartum. Data are from samples taken at birth (precolostrum; 0 d), 2 d (postcolostrum), 14, 21 and 42 d of age. The *P*-values for the main effect of group (G), time (T), and their interaction (G×T) are shown. Main separation between time points was evaluated when a G×T interaction (P ≤ 0.10) was observed, and differences (*) were declared at P ≤ 0.05. Values are means, and SE are represented by vertical bars.

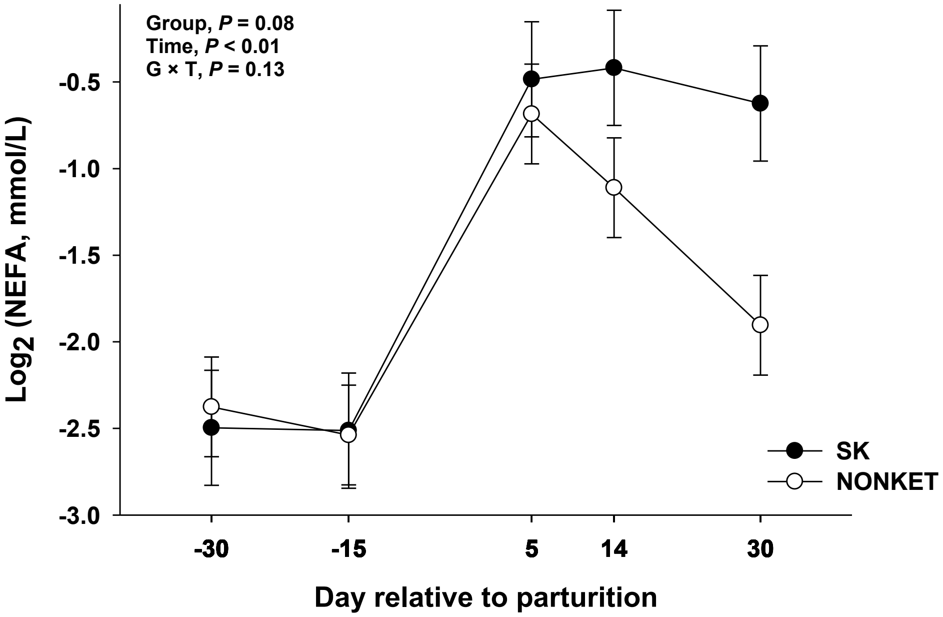

Figure S2. Blood non-esterified fatty acids from cows classified as subclinical ketotic (SK; ≥ 1.4 mmol/L) or nonketotic (NONKET; < 1.4 mmol/L) during the first 10 days postpartum. Data are from samples taken at various time points during the transition period. The *P*-values for the main effect of group (G), time (T), and their interaction (G×T) are shown. Values are means, and SE are represented by vertical bars.

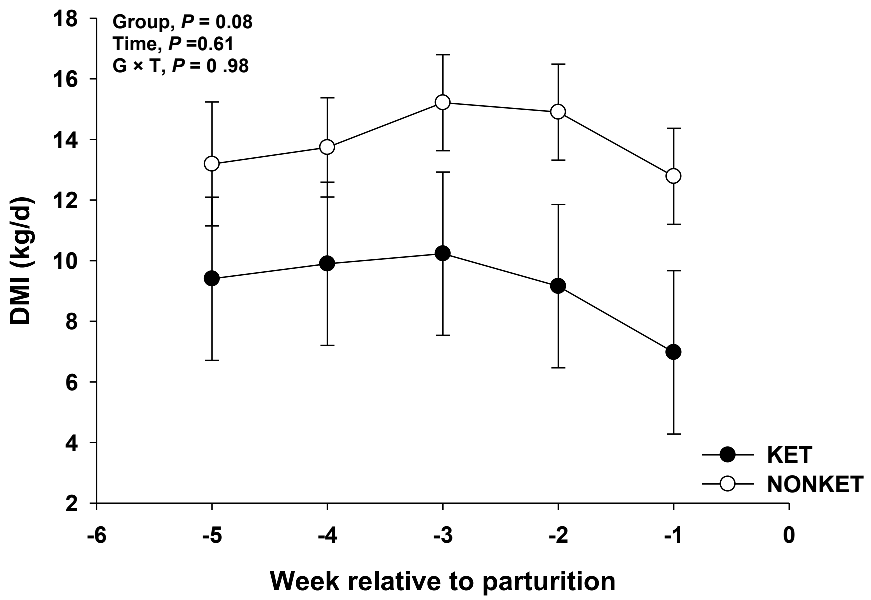

Figure S3. Prepartal dry matter intake from cows classified as subclinical ketotic (SK; ≥ 1.4 mmol/L) or nonketotic (NONKET; < 1.4 mmol/L) during the first 10 days postpartum. The *P*-values for the main effect of group (G), time (T), and their interaction (G×T) are shown. Values are means, and SE are represented by vertical bars.

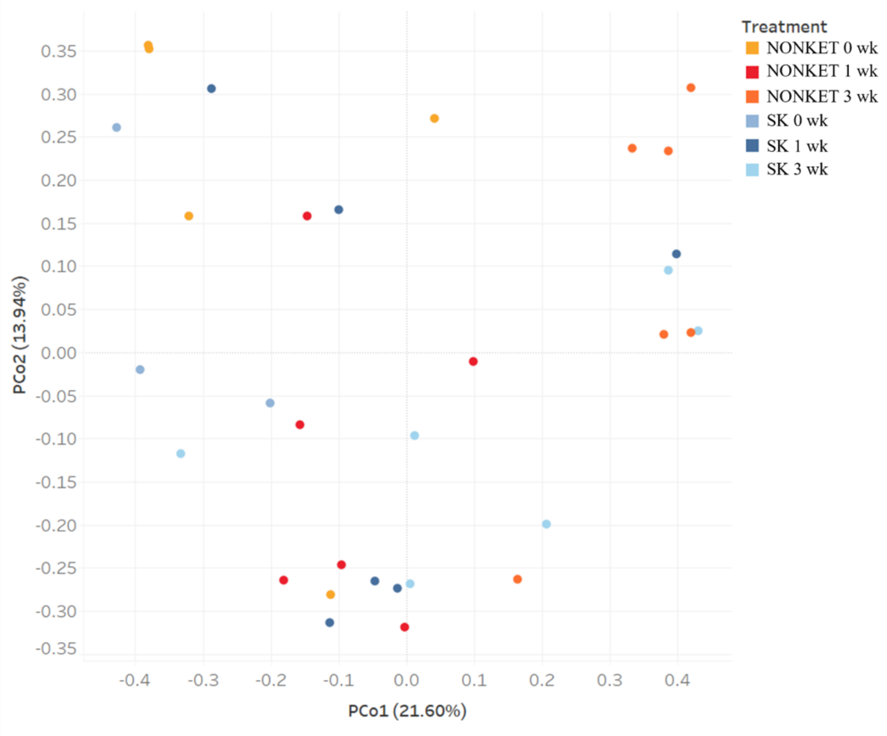

Figure S4. Comparison of fecal bacterial communities in calves born to subclinical ketotic (SK) or non-ketotic (NONKET) cows at 0, 1, and 3 wk of age. PCoA was performed using a Bray-Curtis distance matrix. The x and y axes correspond to Principal Components 1 (PCo1) and 2 (PCo2).
